# Large-Scale Data Analysis of Post-Traumatic Stress Disorder (PTSD) Through GWAS Fine-Mapping and Systems Biology

**DOI:** 10.3390/jpm16080426

**Published:** 2026-08-12

**Authors:** Alireza Sharafshah, Colin Hanna, Kai-Uwe Lewandrowski, Mark S. Gold, Brian Fuehrlein, Panayotis K. Thanos, Igor Elman, Eliot L. Gardner, Jag Khalsa, David Baron, Abdalla Bowirrat, Albert Pinhasov, Edward J. Modestino, Rossano Kepler Alvim Fiorelli, Sergio L. Schmidt, Morgan P. Lorio, Keerthy Sunder, Lyle Fried, Michael Slifer, Frank Fornari, Shaurya Mahajan, Yatharth Mahajan, Marco Lindenau, Álvaro Dowling, Rafaela Dowling, João Paulo Bergamaschi, Kyriaki Z. Thanos, Paul R. Carney, Kenneth Blum

**Affiliations:** 1 Post-Graduate Program in Neurology, Federal University of the State of Rio de Janeiro, Rio de Janeiro 22290-240, Brazil; business@tucsonspine.com (K.-U.L.); fiorellirossano@hotmail.com (R.K.A.F.); slschmidt@terra.com.br (S.L.S.); 2 Division of Personalized Neurobiology & Genetics, The Origen Scientific Institute, Austin, TX 78701, USA; jkhalsa@yahoo.com (J.K.); edward.modestino@gmail.com (E.J.M.); mloriomd@gmail.com (M.P.L.); sunderkr@gmail.com (K.S.); ffornari@me.com (F.F.); shaurya.mahajan1013@gmail.com (S.M.); yatharth.mahajan1226@gmail.com (Y.M.); marco.lindenau@yahoo.com (M.L.); adowling@dws.cl (Á.D.); rafaeladowling@gmail.com (R.D.); jberga@clinicaatualli.com.br (J.P.B.); 3 Department of Psychology, University South Dakota, Vermillion, SD 57069, USA; 4 Department of Orthopaedics, Fundación Universitaria Sanitas, Bogotá 111321, Colombia; 5 Division Personalized Pain Research and Education, Center for Advanced Spine Care of Southern Arizona, Tucson, AZ 85712, USA; 6 Department of Psychiatry, Washington University School of Medicine, St. Louis, MO 63110, USA; mgold9876@wustl.edu; 7 Department of Psychiatry, Yale University School of Medicine, New Haven, CT 06511, USA; brian.fuehrlein@va.gov; 8 Behavioral Neuropharmacology and Neuroimaging Laboratory on Addictions, Clinical Research Institute on Addictions, Department of Pharmacology and Toxicology, Jacobs School of Medicine and Biosciences, State University of New York at Buffalo, Buffalo, NY 14260, USA; 9 Department of Molecular Biology, Adelson School of Medicine, Ariel University, Ariel 4070000, Israel; dr.igorelman@gmail.com (I.E.); bowirrat@gmail.com (A.B.); albertpi@ariel.ac.il (A.P.); 10 Department of Psychiatry, Harvard University School of Medicine, Cambridge, MA 02115, USA; 11 Department of Psychiatry, John Hopkins University School of Medicine, Baltimore, MD 21287, USA; egardner@intra.nida.nih.gov; 12 Department of Microbiology, Immunology, and Tropical Medicine, The George Washington University School of Medicine, Washington, DC 20037, USA; 13 Center for Sports, Exercise, Mental Health, Western University Health Sciences, Lebanon, OR 91766, USA; dbaron@westernu.edu; 14 Department of Psychiatry & Behavioral Sciences, Stanford University, Palo Alto, CA 94305, USA; 15 Brain & Behavior Laboratory, Department of Psychology, Curry College, Milton, MA 02186, USA; 16 Orlando College of Osteopathic Medicine, Winter Garden, FL 34787, USA; 17 Karma Doctors & Karma TMS, and Suder Foundation, Palm Springs, CA 92264, USA; 18 Department of Medicine, Riverside School of Medicine, University of California, Riverside, CA 92521, USA; 19 Division of Personalized Psychiatry, Beach House Center for Recovery, Juno Beach, FL 33408, USA; lylefried@bellsouth.net (L.F.); mslifer@med.miami.edu (M.S.); 20 Department of Psychology, University of Buffalo, Buffalo, NY 14068, USA; kyriaki.thanos@buffalo.edu; 21 Departments of Pediatrics and Neurology, University of Missouri School of Medicine, Columbia, MO 65212, USA; paul.carney1@gmail.com

**Keywords:** PTSD, GWAS, fine-mapping, LD, haplotype, PGx

## Abstract

**Background/Objectives**: Post-Traumatic Stress Disorder (PTSD) is a complex psychiatric condition with a strong polygenic and stress-related biological basis. Although Genome-Wide Association Studies (GWAS) have acknowledged abundant risk variants, translating these findings into biologically meaningful candidates remains challenging. This study introduces an integrative computational approach from raw file preparation by python-coded application into downstream in-depth silico analyses designed to systematically refine GWAS signals for PTSD using fine-mapping, linkage disequilibrium (LD), and haplotype analyses. **Methods**: GWAS source file for PTSD was obtained from the GWAS Catalog (EFO_0001358) and analyzed using a custom Python pipeline integrating data harmonization, genome-wide visualization, LD estimation via 1000 Genomes reference panels, approximate Bayesian fine-mapping, and Haploview-inspired haplotype inference. SNPs were filtered based on statistical significance, LD structure, and posterior inclusion probability. Downstream systems’ biology analyses included protein–protein interaction modeling and pharmacogenomics (PGx) annotations. **Results**: From the primary GWAS dataset, 100 top-ranked SNPs were selected, leading to the identification of 58 significant loci. LD and haplotype analyses refined these signals to 93 candidate SNPs (66 genes). Following the exclusion of non-protein-coding genes, 45 genes remained, and network-based prioritization generated a final list of 20 biologically connected and pharmacogenetically relevant genes associated with PTSD. **Conclusions**: This integrative approach provided a robust and reproducible framework for GWAS fine-mapping and variant prioritization, effectively reducing large-scale GWAS outputs to biologically interpretable PTSD risk loci and genes.

## 1. Introduction

### 1.1. Background

Following nearly four decades of gene-based research in association with finding and characterizing addiction-related Reward Deficiency Syndrome (RDS), the present article introduces a novel evidence-based model for preventing and treating all types of addictive behaviors, not limited to those having known genetic reasons. It is vital to consider that stress is definitely a primary factor in changing the functionality of a normal brain. The new implementation of “Precision Behavioral Management” (PBM) appears imminent. Undeniably, further work is necessary to identify the most suitable candidate genes linked to addiction-prone risk, but PBM development seems to already be underway. Personalized and precision medicine detects very convergent candidate gene single-nucleotide polymorphisms (SNPs) associated in individuals with RDS [1].

The environment displayed by an epigenetic switch controlling gene expression is thought to account for almost 50% of the genetic risk for several psychological disorders and diseases. Post-Traumatic Stress Disorder (PTSD) is a suitable example of the influence that environmental stress has, which is demonstrated by the compounding of the genetic risk. It is now broadly accepted that persons with PTSD also have hypodopaminergia and high rates of comorbidity with substance use disorders (SUD) [2]. Our research consortium is sure that the scientific community will finally support pioneering research designed for dopamine upregulation in mesolimbic structures, a National Institute on Drug Abuse (NIDA)-directed aim, to restore homeostasis [3].

Blum studied the neurochemical mechanisms of alcohol and opioids, beginning in the 1970s, and, in the mid-1990s, developed the concept of RDS [4]. A total of 1315 articles on Reward Deficiency have been indexed in PubMed, and the RDS concept was included in *The SAGE Encyclopedia of Abnormal and Clinical Psychology* in 2017 [1]. Cross-addictions occur primarily in psychiatric disorders, including PTSD [5]. Cocaine use is common among individuals with PTSD and is associated with treatment failure as well as adverse health and societal outcomes. Cocaine use and abuse were also found to increase the prevalence of PTSD symptoms, particularly in females [6]. This increased risk is not unexpected, as significant cocaine abuse leads to hypodopaminergia, which may further exacerbate many PTSD symptoms like nightmares.

These neurochemical disruptions caused by cocaine are considered “physical” rather than “psychological” addiction, and dopamine depletion associated with CUD has been treated using dopamine agonist therapy [7]. Following a single dose of the potent dopamine D2 agonist bromocriptine, cocaine craving was significantly reduced [8]. Open-label trials at low doses suggested that bromocriptine may be useful in cocaine detoxification and serve as an effective non-addictive pharmacological treatment for CUD. Ernest P. Noble conducted a placebo-controlled study of bromocriptine in subjects with Alcohol Use Disorder (AUD) [9]. The most favorable outcomes were observed in bromocriptine-treated subjects carrying the DRD2 A1 allele (first associated with severe alcoholism by Blum et al. in 1990 [10]), whereas the poorest outcomes were observed in placebo-treated A1 subjects [11]. Unfortunately, chronic administration of this potent D2 agonist induces significant downregulation of D2 receptors, thereby limiting its clinical utility [12]. This finding led Blum et al. to propose that milder therapeutic neuro-nutrient formulations could achieve D2 receptor agonism and help attenuate the effects of stressful events [13].

The Blum group advocated for the promoting of dopamine release through the use of milder therapeutic neuro-nutrient formulations, like KB200, to increase human mRNA expression and promote the proliferation of D2 receptors [14].

In conclusion, when D2 receptor levels increase, the craving for psychoactive substances and even non-drug addictive behaviors decreases. It is noteworthy that the promotion of long-term dopaminergic activation through lower-potency and gentle dopaminergic repletion therapy (KB220Z) has been shown to be a clinically effective approach for treating RDS-related behaviors, including PTSD, SUD, Attention-Deficit/Hyperactivity Disorder (ADHD), and obesity [15]. Regarding KB220Z, increased resting-state functional connectivity and enhanced neuronal recruitment have also been demonstrated acutely with this compound, using fMRI in both animals and humans. In fact, within 15 min in animals and 60 min in humans following administration of the neuro-nutrient therapy, additional dopamine neuronal activation has been observed in the brain regions involved in reward processing, potentially resulting in neuroplastic changes [16,17].

### 1.2. Neuroimaging PTSD in Brief

Continued stress may exert long-lasting impacts on cognition [18]. Animal models suggest that alterations within the mesofrontal circuit involving dopaminergic activation may lead to deficits in executive functioning. Building on these findings, van Wingen and colleagues reported that combat stress reduced midbrain activity and integrity and was associated with an impaired ability to sustain attention. Indeed, van Wingen et al. [19], in their long-term follow-up analysis, revealed that the structural and functional changes had normalized within 1.5 years. However, the reduced functional connectivity between the midbrain and prefrontal cortex persisted.

The role of dopamine as the mesolimbic system neurotransmitter underlying motivational function supports the concept of low-potency dopaminergic repletion therapy [20], while endorphinergic enhancement to balance and induce “dopamine homeostasis” across the brain reward circuitry may represent an epigenetic repair approach [21]. The hypothesis is that non-potent D2 receptor stimulation triggers a feedback mechanism in the mesolimbic system, resulting in increased human mRNA expression and the subsequent proliferation of the D2 receptors. The therapeutic model for PTSD would be PBM: the Genetic Addiction Risk Score (GARS) test for genetic predisposition and customized neuro-nutrient supplementation, based on GARS test results, which enhances endorphinergic function and subsequently augments neuroimmunological function, a prerequisite for PTSD attenuation. Recent findings suggesting that mental health treatment immediately following deployment may mitigate the development of PTSD symptoms indicate that this may be an appropriate period to enhance dopamine levels and reduce symptoms associated with reward deficiency.

### 1.3. Neuroimmunological Function, Adaptation of Stress, and Social Behavior

A series of early studies suggested that the advantageous impacts of T-cells following traumatic CNS injury are mediated by autoreactive T-cells specific to brain antigens [22,23,24]. The immune system (mainly vis T-cells) influences the central nervous system’s (CNS) function in disease and health. Evidence indicates that brain immune cells play a critical role in learning and memory. Primary studies revealed that Severe Combined Immunodeficiency (SCID) mice perform poorly in the Morris water maze and other tasks involving memory and spatial learning [25,26]. Notably, reconstituting the T-cell compartment rescued memory and learning in SCID mice [26].

Additionally, in support of the specific impact of T-cells on learning behavior, it is acknowledged that mice lacking B cells show no signs of damage in learning behavior [27]. Notably, SCID mice display weakened social behavior, as shown in the three-chamber sociability assay [28], which assesses the preference of a test mouse to explore a nre mouse over a nre object [29]. Remarkably, while the team did not know how long the impact took, deficits in social behavior were rescued four weeks following repopulation with wild-type lymphocytes.

Furthermore, in a model of PTSD, mice lacking T-cells also show increased vulnerability [30], poor maternal behavior, and obsessive grooming behaviors that are indicative of reward deficit [31]. According to physiology, it is noteworthy that the meningeal spaces feature a varied immune repertoire devoted to neuroimmune interaction, enabling the brain to function at its best, whereas perivascular macrophages and microglia patrol the brain parenchyma [32,33,34,35].

According to most social scientists, social or aggregation behavior is essential not only for survival and evolution but also for the transmission of infectious organisms. Consequently, coordinated immunity and sickness behavior have evolved as mechanisms to restrict the spread of pathogens. Based on prior reports, sickness behavior is not a maladaptive response to infection, but rather a coordinated behavioral adaptation that enhances host survival [36]. More recently, Starkman et al. linked sickness behavior to altruism and kin selection [37]. Another interesting hypothesis suggests that individuals carrying specific gene polymorphisms may preferentially cluster together with respect to sickness behavior or psychological and psychiatric disorders [38,39]. Supporting this concept, Blum’s group [40] proposed that political, spiritual, and social behaviors may be associated with inherited reward gene polymorphisms similar to those implicated in addictions and PTSD. For instance, both alcohol consumption and obesity appear to aggregate within large social networks, potentially influenced by individuals sharing common genotypes such as the DRD2 A1 allele. Such clustering may provide an evolutionary advantage for both the herd and the broader population. Although multiple polymorphic risk alleles may contribute to the PTSD phenotype, Comings, Muhleman, and Gysin [41] were the first to provide genetic evidence linking the DRD2 A1 allele to PTSD. With regard to the immune response, it is well established that the inflammatory response mediated by TNF-alpha contributes to numerous clinical manifestations associated with autoimmune disorders and psychiatric conditions. Indeed, Petrullj et al. [42] demonstrated that elevated systemic inflammation enhances stimulant-induced striatal dopamine release and is associated with increased TNF-alpha plasma levels. This raises the question of whether enhanced neuroimmunological function combined with increased dopamine activity could attenuate stressful experiences, including trauma. Although this hypothesis requires further investigation in both animal models and human studies, a more practical approach may involve identifying strategies to strengthen the immune response by modulating specific signal transduction pathways through targeting the endorphinergic system.

### 1.4. Endorphins and Enhancement of Immune Function and Stress

β-Endorphins interact with opiate receptors through their N-terminal domain, whereas N-terminal acetylation inhibits this interaction. In contrast, the C-terminal region binds to distinct non-opioid receptors [43]. Notably, accumulating evidence suggests that lymphocytes express receptors for these endogenous opioid peptides (EOPs). Furthermore, naloxone has been shown to block the binding of both Met-enkephalin and morphine to T-cells, indicating the presence of opiate receptors on T-lymphocytes [44]. More specifically, these findings demonstrate that normal human peripheral blood T-lymphocytes possess cell-surface receptor-like structures capable of recognizing morphine, dextromoramide, and methionine-enkephalin. Collectively, these observations support the existence of a functional connection between the central nervous system and the immune system.

In summary, considerable evidence indicates that stress rapidly stimulates the release of β-endorphin from the pituitary gland [45]. Soldiers whose leukocytes expressed higher levels of glucocorticoid receptors (which play a key role in the stress response) were found to be at greater risk of developing PTSD following exposure to traumatic events [46]. Given that both stress and trauma influence immune function, these immune adjustments may be associated with corresponding changes in circulating endorphin levels. Smith, Morrill, Meyer, and Blalock [47] proposed that lymphocytes are capable of synthesizing immunoreactive ACTH and endorphin-like peptides. Importantly, endogenous opioid peptides (EOPs) produced by activated lymphocytes may serve as potent regulators of the ultimate immune effector response. In addition, the immunostimulatory effects induced by opioid peptides may be mediated, at least in part, through the dopaminergic system [48]. The modulatory actions of endorphinergic pathways on the immune system, together with their capacity to enhance immune responses under conditions of stress, provide a strong rationale for developing strategies aimed at epigenetically increasing endorphinergic activity through the inhibition of the enzyme enkephalinase [49].

### 1.5. Raising the Endorphin Function via Enkephalinase Inhibition

The enzyme enkephalinase, a carboxypeptidase, degrades methionine-enkephalin into inactive non-opioid fragments, thereby terminating the biological activity of the parent peptide. The enkephalinergic neuroendocrine–immune regulatory network is recognized as one of the most important neuroendocrine–immune systems in humans. Within this system, the neurotransmitter [Met5]-enkephalin released by neuroendocrine tissues plays a key role in regulating immune system development. Enkephalinase inhibitors act by suppressing the enzymatic activity responsible for the degradation of endogenous enkephalin opioid peptides, thereby prolonging their biological effects. Representative compounds within this class include racecadotril, bestatin, RB-101, D-phenylalanine, opioid peptides, opiorphin, thiorphan, and spinorphin [50].

In a pioneering study conducted in 1987, Blum and colleagues demonstrated that both acute and chronic administration of D-phenylalanine, an enkephalinase inhibitor, significantly reduced voluntary as well as forced ethanol consumption in alcohol-preferring mice characterized by genetically determined deficiencies in brain enkephalin levels. Because enkephalinase inhibition increases endogenous enkephalin concentrations within the brain, the authors proposed that the modulation of endogenous opioid peptide activity may represent an important mechanism for regulating excessive alcohol intake. Given that drug-seeking behavior and PTSD appear to share overlapping neurochemical and genetic mechanisms, and considering the potential role of neuroimmunological regulation in alleviating PTSD symptoms, the strategy of enhancing endorphin function through the inhibition of enkephalinase represents a promising therapeutic concept. Several studies have reported the anti-inflammatory effects of D-phenylalanine in rat paw inflammation models in support of this view [51,52].

Of note, Nagarjun et al. [52] reported in 2017 that a chromium complex of D-phenylalanine [Cr(d-phe)3] exerted protective effects against indomethacin-induced Irritable Bowel Disorder (IBD) in rats. These beneficial effects were attributed to the compound’s anti-inflammatory and antioxidant properties. This observation is especially noteworthy in light of findings by Febo, Blum, Badgaiyan, Perez, Colon-Perez, and colleagues [16], who utilized resting-state functional magnetic resonance imaging (rsfMRI) to demonstrate BOLD activation within the dopaminergic pathways of the brain reward circuitry, following administration of KB220Z, which is a nutraceutical formulation containing both chromium and D-phenylalanine.

### 1.6. Understanding Genetics of PTSD

As noted previously, David Comings conducted the first study demonstrating an association between a reward-related gene, the dopamine D2 receptor gene (DRD2; A1 allele), and PTSD in military veterans [41]. Individuals carrying this allele (which was previously linked to severe alcoholism by the Noble–Blum group) possess approximately 30–40% fewer dopamine D2 receptors in the brain [53].

Consistent with this observation, reduced dopaminergic function has been associated with an increased susceptibility to PTSD [21]. During exposure to extreme combat-related stress, dopamine release from neurons may increase to levels approaching 100-fold above the baseline in an attempt to cope with the physiological demands of stress [54,55,56]. Such excessive dopaminergic activation can ultimately lead to dopamine depletion. When combined with trait hypodopaminergia (characterized by reduced D2 receptor density) or repeated exposure to severe stressors, this depletion may contribute to the development of PTSD. Evidence further supports a heritable component to PTSD. Studies of twins have shown that monozygotic twins exhibit a substantially greater concordance for PTSD than dizygotic twins, suggesting that genetic factors influence vulnerability to the disorder following combat exposure, including service in the Vietnam War [57].

In addition, individuals with relatively smaller hippocampal volumes—a brain region critically involved in memory processing—appear to be more susceptible to developing PTSD after traumatic experiences, potentially reflecting underlying genetic influences [58]. PTSD also shares a considerable proportion of its genetic architecture with other psychiatric disorders. For example, approximately 60% of the genetic variance underlying panic disorder and generalized anxiety disorder overlaps with PTSD, while substance use disorders exhibit more than 40% genetic similarity [59].

Importantly, genetic predisposition alone does not fully explain PTSD susceptibility. Environmental exposures, particularly childhood adversity and abuse, can induce epigenetic modifications that alter brain function and neurochemical signaling. Unlike DNA sequence variations, epigenetic changes involve environmentally driven alterations in gene regulation that affect cellular, physiological, and behavioral phenotypes. Of particular concern, these modifications may persist across multiple generations [60]. Environmentally induced alterations in chromatin structure can reduce dopamine D2 receptor expression and function, thereby increasing vulnerability to PTSD.

Genetic polymorphisms regulating gene expression in the dopaminergic and serotonergic pathways have been implicated in PTSD pathogenesis. One of the most extensively studied loci is 5-HTTLPR, located within the promoter region of SLC6A4, the gene encoding the serotonin transporter. Variants within this region influence emotional responses to traumatic experiences and, when combined with environmental stressors, may increase the likelihood of both depression and PTSD. Within the dopaminergic pathway, the DRD2 A1 allele has been associated not only with PTSD, but also with numerous reward deficiency-related behaviors and comorbid conditions. Additional candidate genes have also been implicated. Variants in GABRA2 have been associated with PTSD risk, while the interaction between traumatic exposure and the Val158Met polymorphism of the catechol-O-methyltransferase (COMT) gene has been well documented [2]. Furthermore, polymorphisms within FKBP5 (a co-chaperone of HSP90 that regulates glucocorticoid receptor function) found to influence PTSD susceptibility via gene–environment interactions [61].

Further evidence for the involvement of dopaminergic signaling comes from the work of Zhang, Wang, Cao, Li, Fang, and colleagues who identified a significant interaction between *DRD2/ANKK1* and *COMT* variants (rs1800497 × rs6269) associated with PTSD diagnosis (*p*-corrected = 0.0169155). Both single-variant and haplotype analyses demonstrated that rs1800497 modified the association patterns of the rs6269 G allele as well as the rs6269-rs4633-rs4818-rs4680 G-C-G-G haplotype. Importantly, this interaction was independently replicated in a cohort of young Chinese women (32 cases and 581 controls; *p*-interaction = 0.01329). The findings suggest that rs1800497 influences dopamine D2 receptor density, whereas COMT haplotypes affect catechol-O-methyltransferase expression and enzymatic activity [62].

The identification of military personnel carrying high-risk genetic variants represents a potentially valuable application of the Genetic Addiction Risk Severity (GARS) test. Among the polymorphisms included in GARS, the DRD2 A1 allele has consistently demonstrated an association with both PTSD and comorbid substance use disorders [41]. In an addiction treatment cohort, 58.3% of individuals meeting the diagnostic criteria for combat-related PTSD following service in Vietnam carried the D2A1 allele, compared with only 12.5% of individuals without PTSD (*p* < 0.04). These findings were subsequently replicated, with 61.5% of PTSD patients carrying the D2A1 allele, whereas none of the non-PTSD controls possessed the variant (*p* < 0.002). When both cohorts were combined, the allele was present in 59.5% of PTSD cases but only 5.3% of the controls (*p* < 0.0001) [41]. Collectively, these findings indicate that DRD2 variants in linkage disequilibrium with the D2A1 allele may confer increased susceptibility to PTSD, while the absence of these variants may provide a degree of relative resilience to the disorder.

### 1.7. Can We Prevent or Treat PTSD

Galatzer-Levy et al. [63] applied latent growth mixture modeling (LGMM) together with supervised machine learning (ML) approaches to classify individuals into distinct symptom progression trajectories following trauma exposure. Using trajectory-based and graphical analytical methods, the authors examined causal relationships within a large longitudinal dataset that assessed neuroendocrine responses to traumatic events. Endocrine biomarkers collected in the emergency room (ER), along with PTSD status evaluated at 10 days, 1 month, and 5 months post-trauma, were incorporated into the analysis. Their findings revealed that diminished neuroendocrine responses in the ER were associated with a history of early childhood trauma exposure. This observation is consistent with experimental evidence demonstrating that early-life adversity produces a blunted cortisol response to subsequent traumatic stress in animal models. The authors proposed that these abnormal stress-response patterns may reflect underlying genomic and molecular mechanisms that warrant further investigation. Moreover, atypical cortisol responses appear to be influenced by multiple genetic, epigenetic, and proteomic factors, including FKBP5 among others, potentially representing actionable therapeutic targets in traumatic emergencies such as those encountered by military personnel deployed to combat environments.

The early identification of individuals at elevated risk for PTSD could provide substantial preventive benefits, particularly if implemented within military settings such as the Department of Defense (DOD). Although expanded access to cognitive behavioral therapy and trauma-focused interventions has demonstrated some therapeutic benefit, overall effectiveness remains modest in many patients with PTSD [64]. Another proposed preventive strategy is Critical Incident Stress Management (CISM), which is an integrated and multi-component framework of psychological interventions designed for use during acute adversity, trauma, and disaster situations as needed. CISM has been utilized as both a preventive and therapeutic approach for acute stress disorder, PTSD, post-traumatic depression, bereavement, and grief-related conditions [65]. Current recommendations of the World Health Organization (WHO) discourage the routine use of benzodiazepines and antidepressants immediately following traumatic exposure. In contrast, accumulating evidence supports the use of the anti-inflammatory agent hydrocortisone as a preventive intervention in adults, a concept that aligns with the therapeutic rationale proposed in this review. By comparison, there is limited evidence supporting the effectiveness of other pharmacological agents such as propranolol, escitalopram, temazepam, and gabapentin. Notably, because gabapentin enhances GABAergic neurotransmission while reducing dopaminergic activity, its long-term use may not be optimal for PTSD management [66].

In light of these observations, our laboratory has proposed the use of the pro-dopamine regulator KB220Z as a novel therapeutic approach. Studies conducted by Thomas McLaughlin and colleagues demonstrated that chronic administration of the nutraceutical KB220Z eliminated severe lucid nightmares in approximately 87% of patients diagnosed with PTSD and comorbid ADHD [67,68]. Importantly, improvement in nightmare severity appeared to be dependent upon continued KB220Z administration. Voluntary discontinuation of the compound was frequently followed by the recurrence of distressing dreams. Many patients reported a gradual reduction, followed by the complete resolution, in long-standing terrifying nightmares while receiving KB220Z treatment [69]. Remarkably, in at least four documented cases, the beneficial effects on nightmares persisted for up to 12 months following self-discontinuation of KB220Z [67]. Experimental findings in rodents have further shown that KB220Z increases functional connectivity volume and enhances the recruitment of dopaminergic neuronal activity within brain reward circuitry. These observations suggest the induction of neuroplastic adaptations, potentially mediated through epigenetic mechanisms. Such neuroplastic changes may contribute to the sustained reduction of lucid dreaming and nightmare-related symptoms in PTSD patients [69]. 

Thanks to Genome-Wide Association Studies (GWAS), large-scale analyses verified the inter-individual genetic differences (SNPs) widely associated with PTSD dispositions. In this regard, Ashley-Koch et al. performed GWAS of PTSD in 2312 Iraq–Afghanistan-era veterans and reported the most significant genes, including *UNC13C*, *DSCAM*, *TBC1D2, SDC2*, *PCDH7*, *PRKG1*, and *DDX60L* [70]. Recently, Nievergelt et al. conducted a multi-ancestry meta-analysis of GWAS across 1,222,882 individuals of European ancestry and 58,051 admixed individuals with African and Native American ancestries. They found 95 genome-wide statistically significant loci (80 new loci). They finally found top genes influenced by stress-, immune-, fear-, and threat-related mechanisms, which were formerly hypothesized as to be underlying the neurobiology of PTSD. These findings can support the potential of neurobiological systems linked to PTSD pathophysiology [71]. With this background, our laboratory embarked on a GWAS large-scale analysis involving the integration of fine-mapping and systems biology.

## 2. Material and Methods

### 2.1. Large-Scale Data Analysis (GWAS Fine-Mapping and Systems Biology)

**Figure 1:**
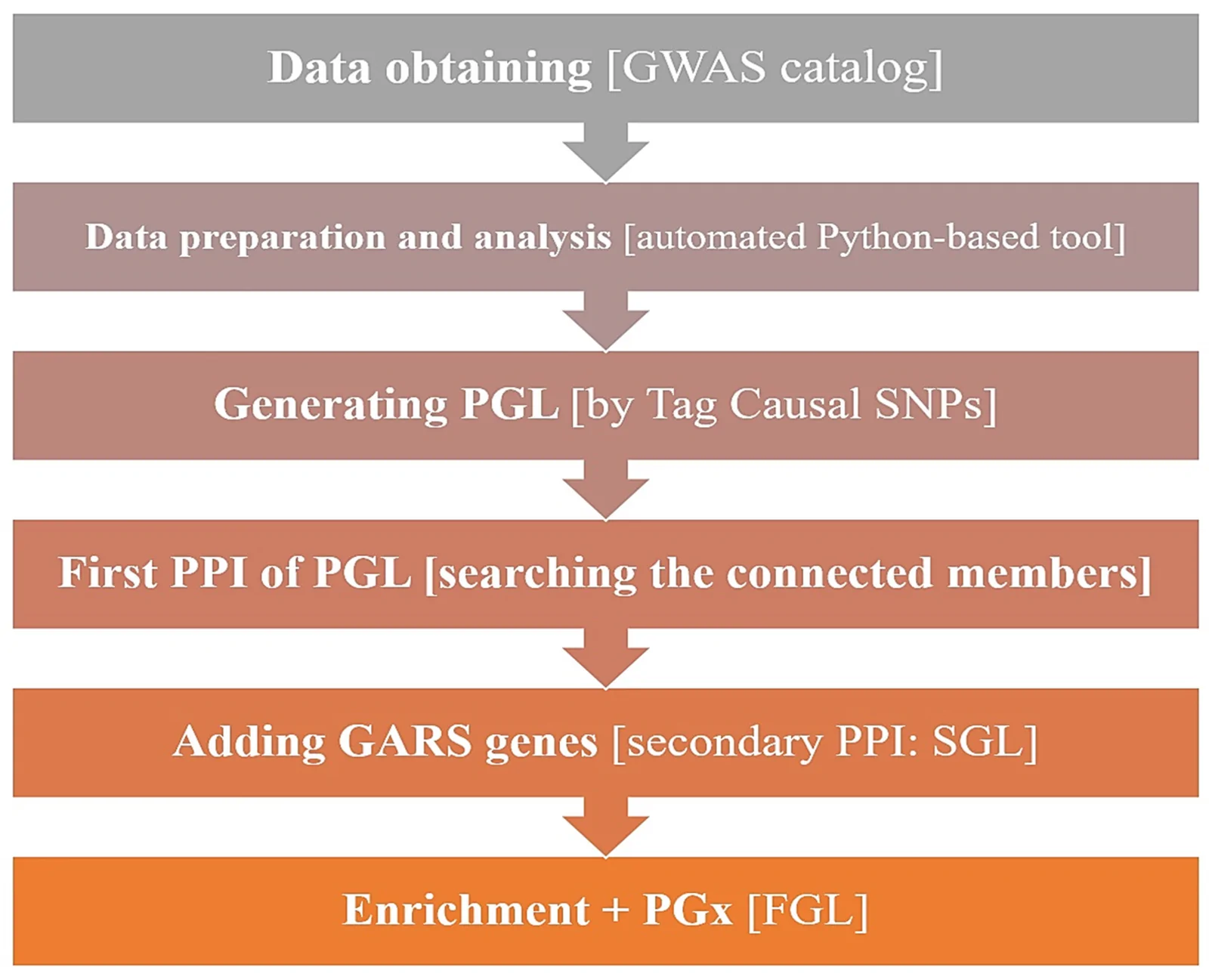
All main steps are summarized and visualized in this flowchart, which indicates the logics and strategy of analysis in a glimpse. PGL, SGL, and FGL are the Primary Gene List, Secondary Gene List, and Final Gene List, respectively.

To have a comprehensive overview on PTSD, we utilized large-scale data analysis through GWAS fine-mapping technique and additional systems biology (Figure 1). In the first step, we employed a newly automated tool, coded by Python, which can analyze a GWAS file (TSV/Excel) through a rationale framework of GWAS fine-mapping. We obtained the source file from the GWAS catalog (https://www.ebi.ac.uk/gwas/ (accessed on 23 December 2025)) with ID: EFO_0001358 and uploaded it to a user interface (UI). Analyses were implemented in Python (ver. 3.13) using Pandas and NumPy for data handling, SciPy for statistical utilities, Plotly (Express/Graph Objects) (ver. 3.11.1) and Matplotlib (v6.6.0) for visualization, and Streamlit for an interactive analysis interface; external LD and annotation queries were performed via HTTP requests (Requests) to Ensembl and (optionally) GTEx APIs (https://www.gtexportal.org). The source file contained various GWAS reports on multiple ancestries accomplished on PTSD as the trait, including different information, among them: author name, year rsID, context, mapped gene, *p*-value, Odds Ratio (OR) or Beta, CI 95%, initial sample size, and other details. The input table was harmonized to retain single-nucleotide polymorphisms (SNPs) with valid rsIDs, chromosomal coordinates (chromosome ID and position), and association *p*-values. Variants with missing genomic position or malformed identifiers were excluded prior to downstream analyses. In the next, a Primary Gene List (PGL) was generated by extracting tag causal SNPs, which, in turn, resulted from the fine-mapping algorithms (such as genetic associations in haplotype and LD blocks). It should be noted that the PGL was an interpretation of SNPs to their mapped genes on Genome browsers such as Ensembl. Then, to prioritize the genes in PGL, we conducted a protein-based network by STRING-MODEL in protein–protein interactions (PPIs) and, by adding GARS genes to the connected members of PGL, we tested the new list by STRING-MODEL again. The results generated the Secondary Gene List (SGL), and further enrichment and PGx analyses on this list uncovered another list resulted from the potential members inside SGL, which is called the Final Gene List (FGL).

### 2.2. Statistical Details

Reported *p*-values were considered in a precise process to accommodate heterogeneous GWAS Catalog formats (e.g., scientific notation and textual representations). For variants with reported ORs and confidence intervals (CIs), log odds effect sizes and standard errors (SE) were inferred when possible; otherwise, SEs were approximated by Wald inversion using the association *p*-value. Duplicate rsIDs were focused by retaining the most significant association signal per variant.

Genome-wide visualization was considered in multiple plots, including Manhattan plot, Quantile–Quantile (QQ) plot, and heatmap plot. A Manhattan plot was generated by −log_10_ (*p*-value) across genomic coordinates to envisage the overall association perspective. Manhattan plots were configured to label the top 5 significant SNPs by default. Chromosome-wise offsets were applied to preserve genomic ordering, and the conventional genome-wide significance threshold (*p* ≤ 5 × 10^−8^) was indicated. A QQ plot was constructed to assess the deviation from the null distribution of test statistics and, also, to evaluate the potential inflation or deflation of association signals. Genome-wide significant SNPs were clustered into loci based on physical proximity along each chromosome. Significant SNPs were clustered together into a single locus if the gap between them is ≤500 kb. Variants separated by more than a predefined genomic gap were assigned to distinct loci, and each locus was extended by flanking regions to capture local LD structure. A padding of 25 kb is added to both sides of the clustered locus boundary. For each locus, the lead SNP was defined as the variant with the smallest *p*-value. The default threshold for assigning SNPs to an LD block was r^2^ ≥ 0.8. The query window for calculating pairwise LD was capped at 500 kb (matching the Haploview default scale). A maximum of 200 significant SNPs per locus are processed for LD calculations to prevent API timeouts.

Notably, to overcome the lack of having genotypic data for each sample, pairwise LD information (r^2^ and D’) was obtained from the Ensembl REST API using 1000 Genomes Phase 3 reference populations. More specifically, the target population was European as default population (1000GENOMES:phase_3:EUR), with a fallback to the global dataset (1000GENOMES:phase_3:ALL) if the specific population was unselected or unavailable. LD was estimated at the regional level without access to individual-level genotype data. Within each locus, contiguous LD blocks were estimated by an r^2^ threshold, and representative tag SNPs were selected per block according to statistical significance and, where available, posterior inclusion probability (PIP). Approximate Bayesian fine-mapping was performed within loci using Wakefield’s approximate Bayes factor framework when ES estimates and SEs were available. PIPs were calculated on a per-locus basis and used to define approximate 95% credible sets. An additional parameter setting was Primary SNP Checkpoint: the pipeline extracted the top 50 most-significant SNPs for initial checkpoint tracking.

Locus prioritization and visualization were the next steps and were successfully done by the tool. For the top loci, LD matrices were visualized as heatmaps, and haplotype block structures were inferred using a Haploview-inspired “solid spine of LD” heuristic based on D’. Loci were ranked by a composite score integrating the number of significant SNPs, LD strength, and fine-mapping support, and the top five loci were selected for detailed LD and haplotype visualization.

## 3. Results

### 3.1. GWAS Fine-Mapping Results

The program was manually set for the top 100 unique SNPs to be included in the following analysis, and the rationale behind this were the prior observational experiences that resulted from finding the remaining SNPs with *p*-values lower than the 5 × 10^−8^ threshold. To check this and capture any missed SNPs potential to be included for the next step, we set the program for the top 500 SNPs, but the results were the same. Accordingly, 19 SNPs were in chromosome 7, 14 SNPs were located in chromosome 1, 13 SNPs in chromosome 6, 9 SNPs in chromosome 2, 9 SNPs in chromosome 3, 8 SNPs in chromosome 11, 6 SNPs in chromosome 5, 6 SNPs in chromosome 17, 4 SNPs in chromosome 14, 4 SNPs in chromosome 18, 3 SNPs in chromosome 9, 2 SNPs in chromosome 12, 2 SNPs in chromosome 15, and 1 SNP in chromosome 8.

**Figure 2:**
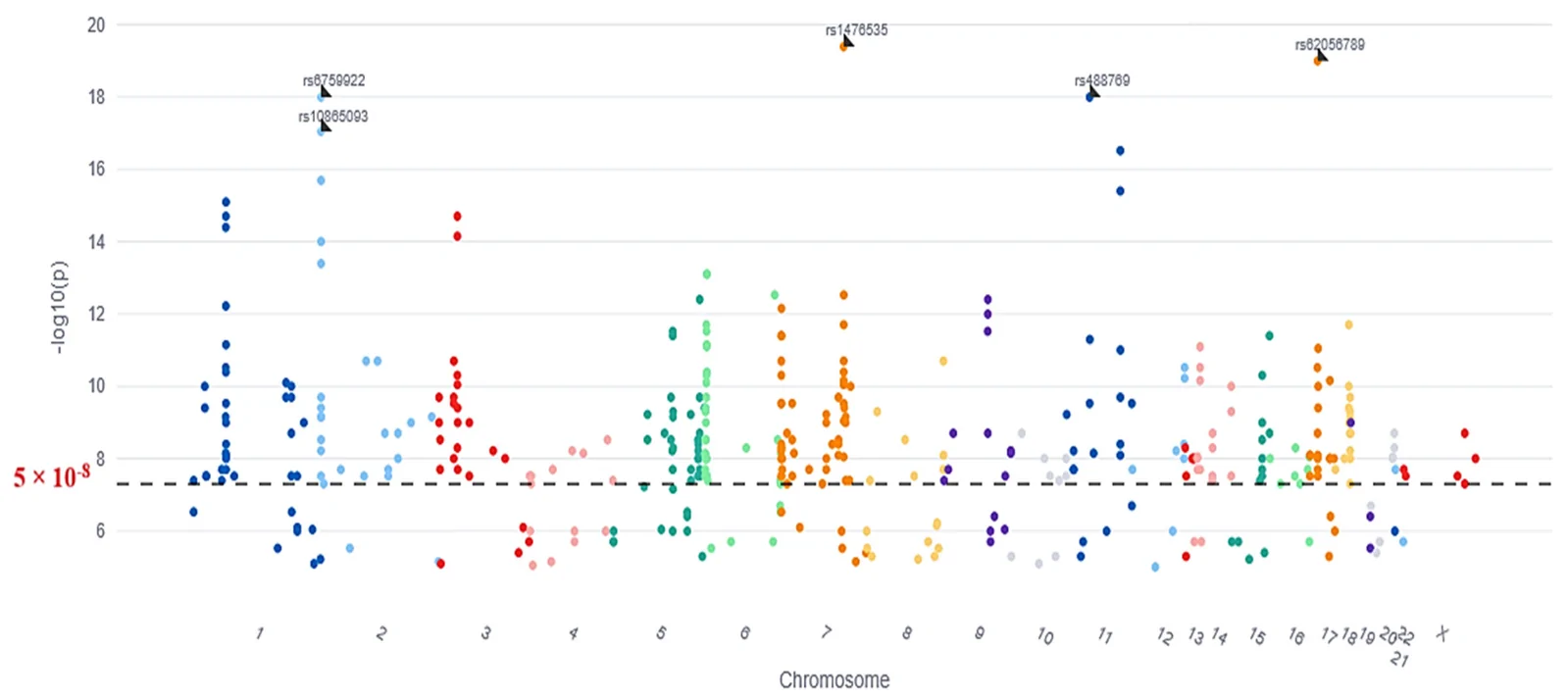
Manhattan plot clustering the rsIDs based on their genomic regions (chrIDs) and −log10 (*p*), with an emphasis on the *p*-value of the 5 × 10^−8^ GWAS consensus threshold.

**Figure 3:**
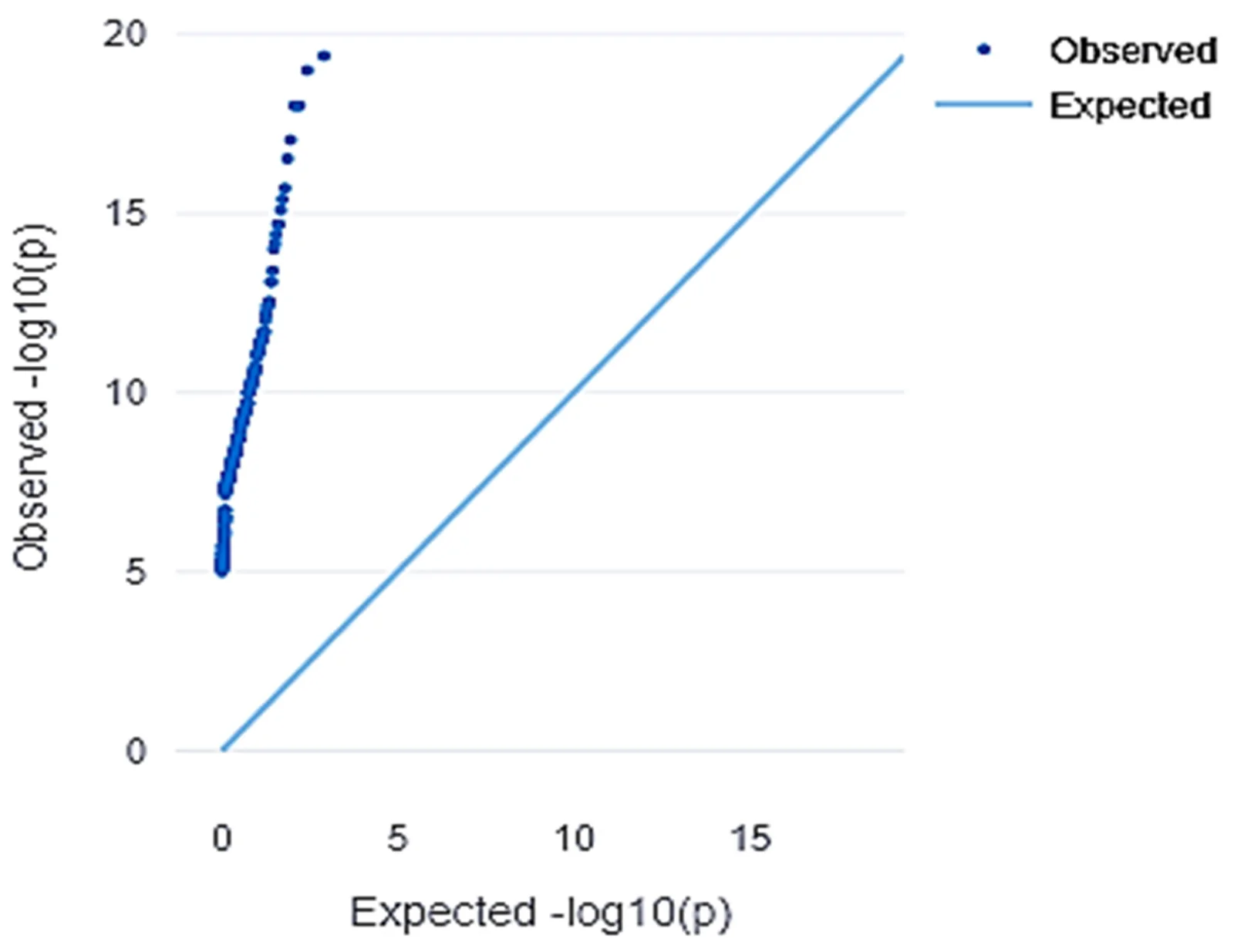
QQ plot indicates the deviation from the expected prediction threshold, which reflects the polygenic associations among the results of dataset analyzed by GWAS fine-mapping procedures.

The Manhattan plot demonstrated a clear clustering of association signals at discrete genomic regions (Figure 2), while the QQ plot (Figure 3) showed deviation from the null expectation in the tail of the distribution, consistent with true polygenic associations rather than systematic inflation.

Clustering of genome-wide significant variants uncovered several independent loci across the genome. Each locus was characterized by a lead SNP with strong statistical support and varying degrees of local SNP density, suggesting heterogeneity in regional LD architecture. The results of the genome-wide visualization of the EFO_0001358 GWAS revealed 58 loci with more than one included SNP which passed the genome-wide significance threshold; among them, chr1: 72792008-73602245 and chr6: 28542056-29659940, which both contained 16 SNPs, were the loci with the highest number of SNPs.

Reference-panel-based LD analysis resolved multiple loci into one or more contiguous LD blocks. Within these blocks, tag SNPs were selected to represent correlated variant groups. Approximate fine-mapping highlighted a subset of SNPs with elevated PIPs, indicating likely causal candidates under a single-causal-variant assumption per locus. The five highest-priority loci displayed distinct LD and haplotype patterns. Some loci showed compact LD blocks with a single dominant signal, whereas others exhibited more complex haplotype structures with multiple correlated SNPs. Heatmap visualizations of r^2^ and D’ values supported these differences and provided intuitive insight into locus-specific genetic architecture.

**Figure 4:**
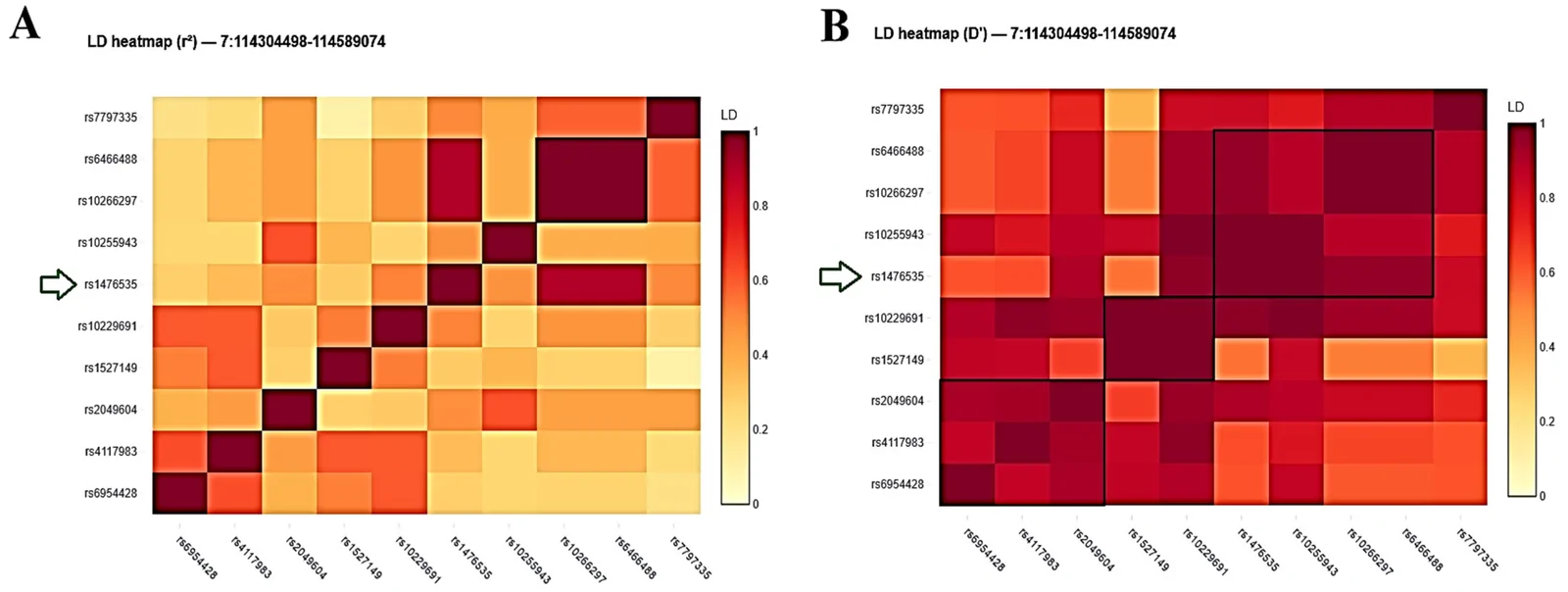
(**A**) LD heatmap and (**B**) Haploview-style haplotype blocks for the chr7: 114304498-114589074 locus based on r^2^ and D’, respectively. The arrows highlight rs1476535 as the lead SNP.

**Figure 5:**
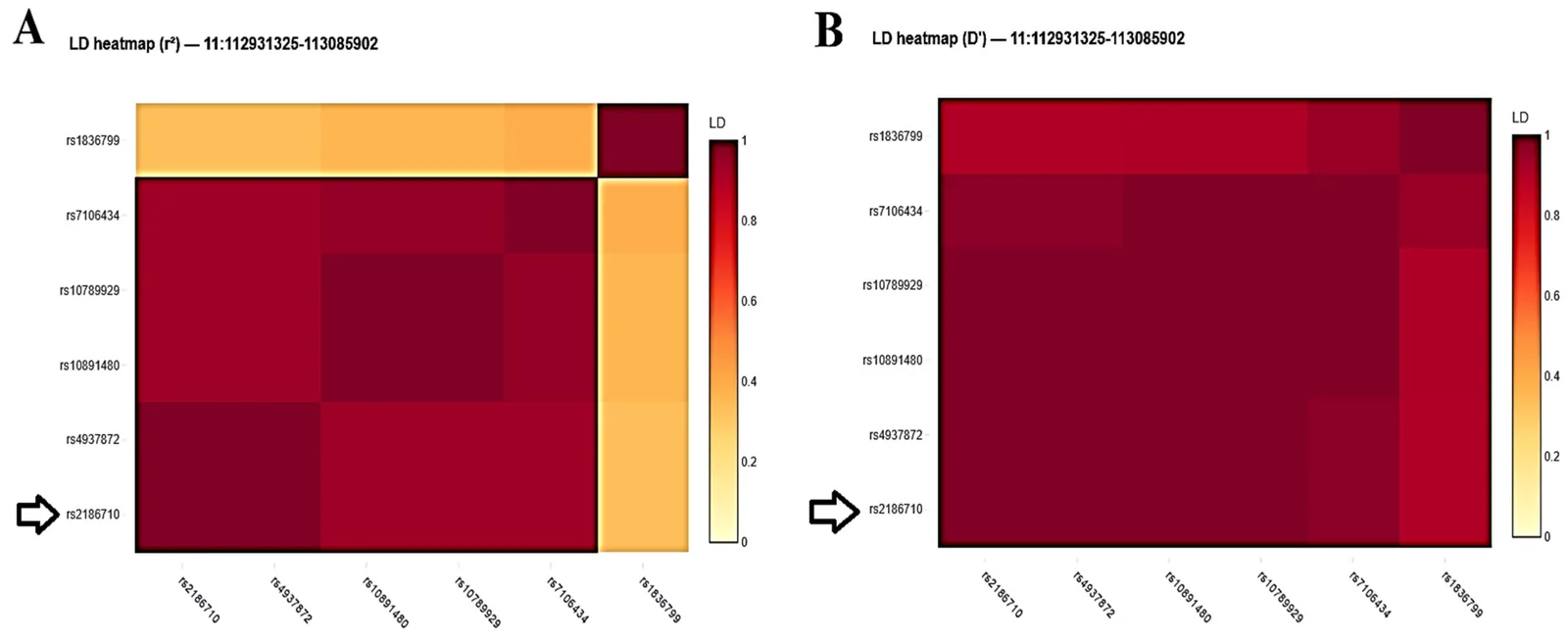
(**A**) LD heatmap and (**B**) Haploview-style haplotype blocks for the chr11: 112931325-113085902 locus based on r^2^ and D’, respectively. The arrows highlight rs2186710 as the lead SNP.

**Figure 6:**
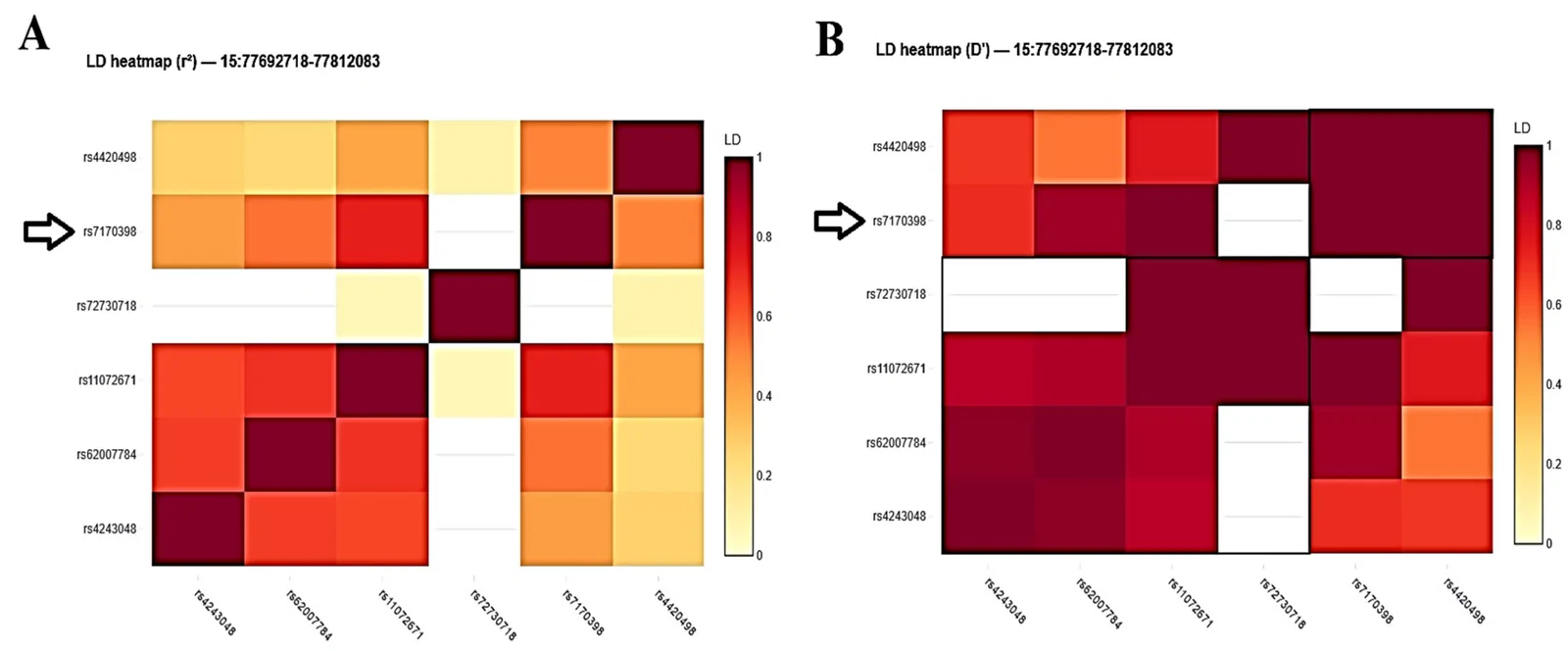
(**A**) LD heatmap based on r^2^ and (**B**) Haploview-style haplotype blocks based on D’ for the chr15:77692718-77812083 locus. The arrows highlight rs7170398 as the lead SNP.

**Figure 7:**
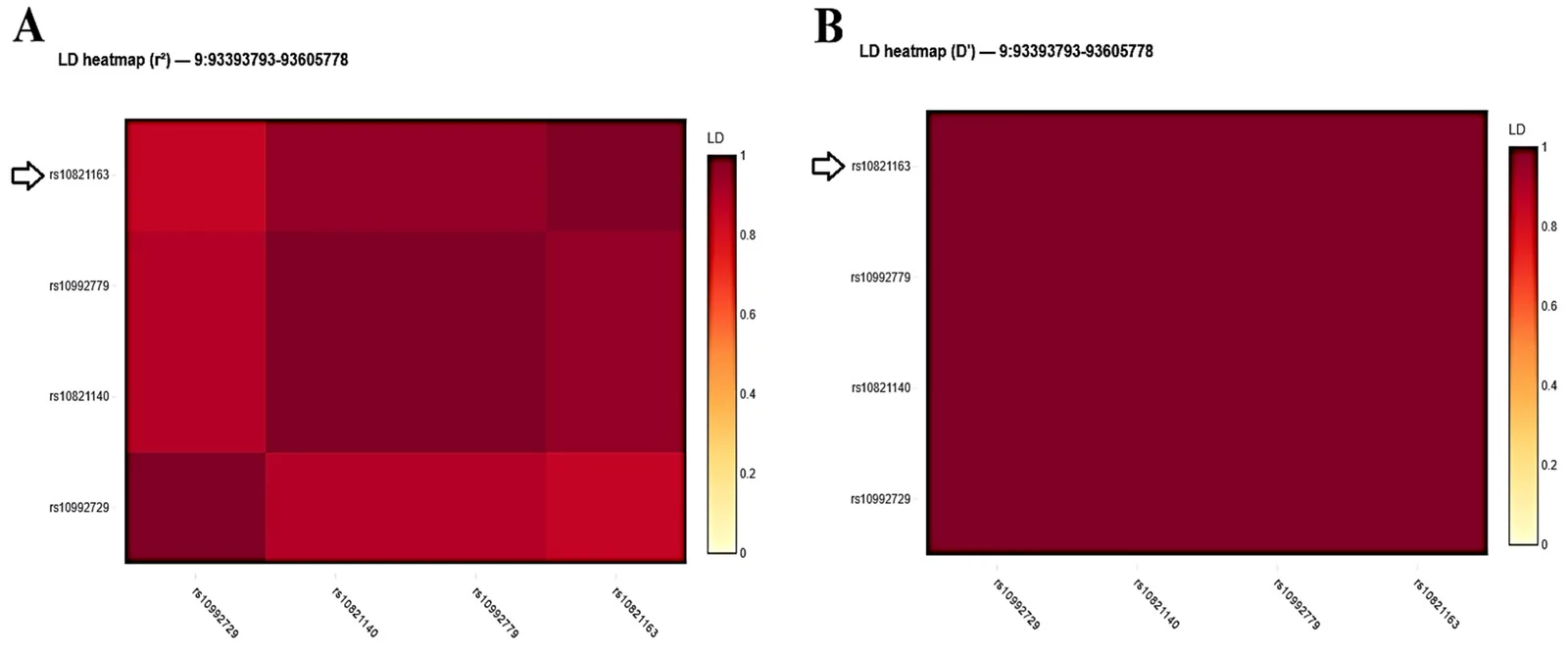
(**A**) Illustrations of LD heatmap based on r^2^ and (**B**) Haploview-style haplotype blocks based on D’ for the chr9: 93393793-93605778 locus. The arrows emphasize rs10821163 as the lead SNP.

**Figure 8:**
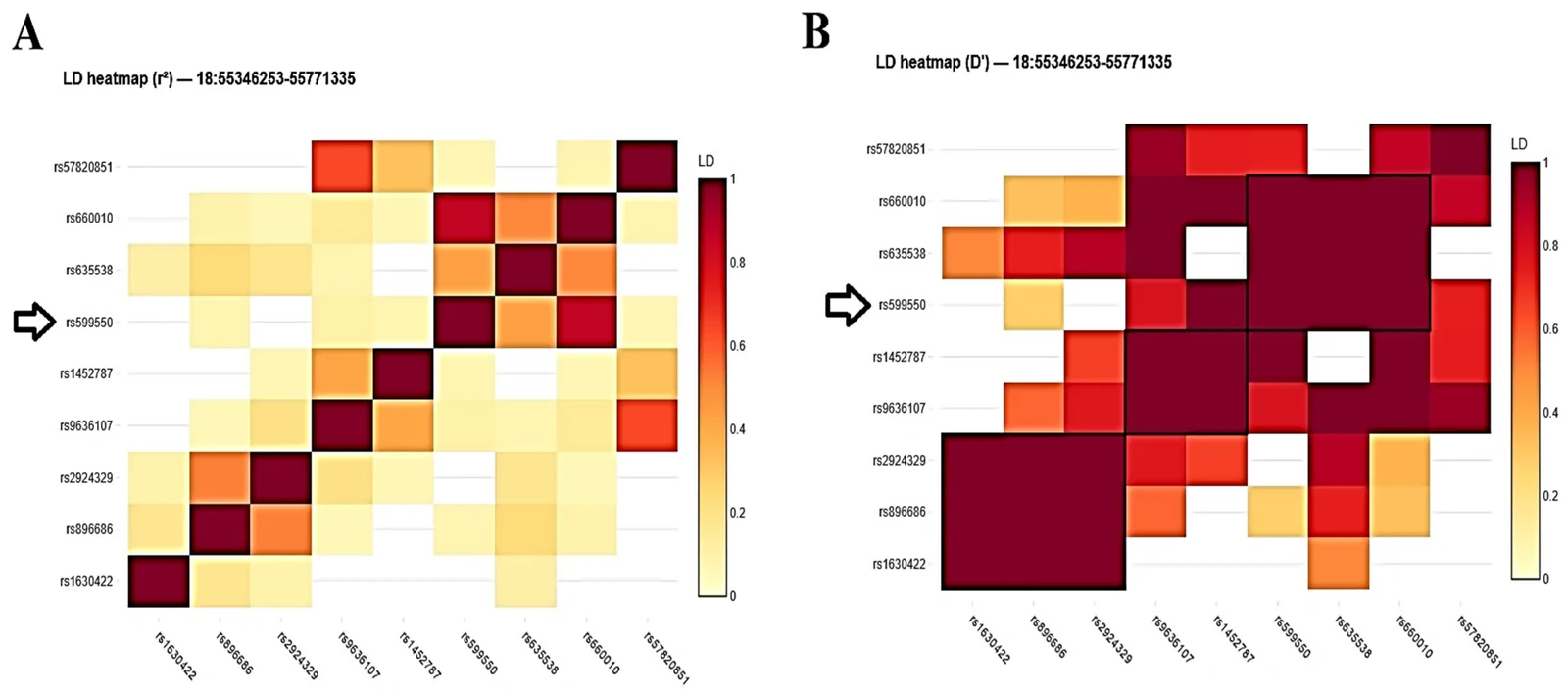
(**A**) Results of LD heatmap based on r^2^ and (**B**) Haploview-style haplotype blocks according to D’ for the chr18: 55346253-55771335 locus. The arrows emphasize rs599550 as the lead SNP.

The final visualizations of LD and Haplotype heatmap plots were performed for the five top loci, including chr7: 114304498-114589074 (containing 10 significant SNPs with rs1476535 as lead SNP) (Figure 4), chr11: 112931325-113085902 (containing 6 significant SNPs with rs2186710 as lead SNP) (Figure 5), chr15: 77692718-77812083 (containing 6 significant SNPs with rs7170398 as lead SNP) (Figure 6), chr9: 93393793-93605778 (containing 4 significant SNPs with rs10821163 as lead SNP) (Figure 7), and chr18: 55346253-55771335 (containing 9 significant SNPs with rs599550 as lead SNP) (Figure 8).

### 3.2. Summary of Candidate Variants

Intersection of primary genome-wide significant SNPs with LD-derived tag SNPs yielded a refined list of 93 candidate SNPs. These SNPs represent a parsimonious set of putative causal signals for EFO_0001358, suitable for downstream functional annotation and integrative analyses. For example, the tag causal SNPs from LD and Haplotype analyses were rs1476535 (*FOXP2*, intronic), rs2186710 (*LINC02763-NCAM1-AS2*, intergenic), rs7170398 (*LINGO1*, intergenic), rs10821163 (*PHF2*, intronic), and rs599550 (*TCF4*, intronic). Accordingly, the Primary Gene List (PGL) generated from the final causal SNP list including 66 mapped gene; among them, FOXP2 was the top gene based on the highest number of Tag causal SNP (8 SNPs). Following the removing of the RNA coding and pseudogenes, 45 protein-coding genes remained (Secondary Gene List: SGL).

### 3.3. Post GWAS Fine-Mapping Analyses

After performing protein–protein interactions (PPIs) by STRING-MODEL [72], five members of the SGL shaped a very little-connected network; thus, we added 10 genes of GARS involved in dopamine homeostasis and Reward Deficiency Syndrome (RDS) to see if they can generate a larger network or not. Surprisingly, 20 connected genes (10 GARS + 10 SGL) were in complete interactions of a larger network compared with the initial network. Finally, we considered the new network as Final Gene List (FGL), including 20 genes: *DRD1*, *DRD2*, *DRD3*, *DRD4*, *OPRM1*, *SLC6A3*, *SLC6A4*, *COMT*, *MAOA*, *GABRA3*, *GABBR1*, *MAD1L1*, *TSNARE1*, *GPX5*, *TCF4*, *LINGO1*, *NCAM1*, *MAPT*, *BPTF*, and *WNT3* (Figure 8).

### 3.4. Pharmacogenetic Searching

**Figure 9:**
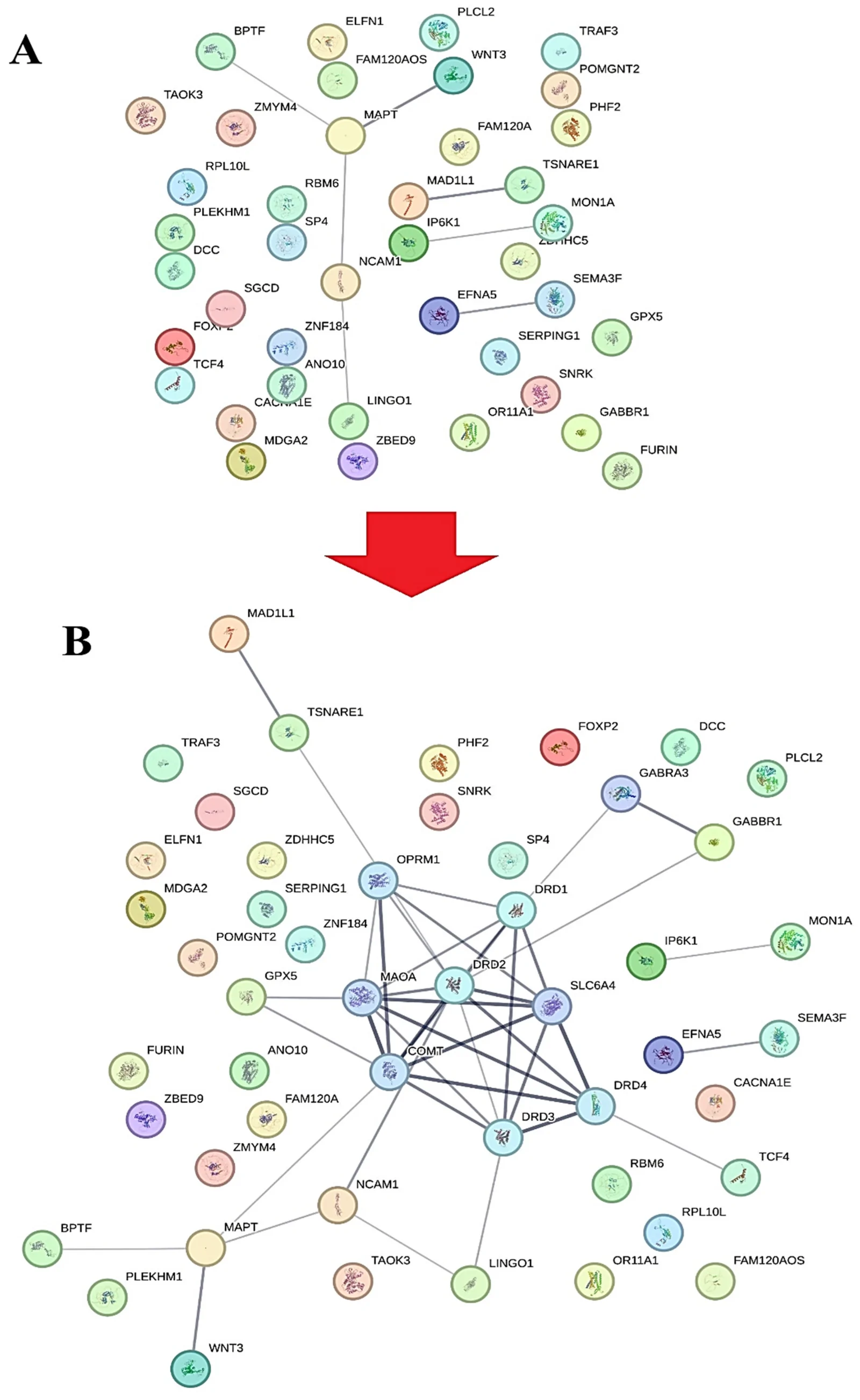
STRING-MODEL of protein–protein interactions (PPIs) in two phases, including phase I when the PGL was derived directly from GWAS fine-mapping analysis (**A**), and phase II when PGL merged with GARS 10 genes (**B**).

To find out more about the potentials of FGL members, we searched each gene in the ClinPGx databank and found genes with at least one PGx annotation: *DRD1*, *DRD2*, *DRD3*, *DRD4*, *OPRM1*, *SLC6A3*, *SLC6A4*, *COMT*, *MAOA*, *GABRA3*, *GABBR1*, *MAD1L1*, *TSNARE1*, *GPX5*, *TCF4*, *LINGO1*, *NCAM1*, *MAPT*, *BPTF*, and *WNT3.* Interestingly, within the aforementioned Final Gene List, GARS genes all have at least one PGx annotation; however, just *MAD1L1*, *GPX5*, and *NCAM1* had a PGx annotation. Furthermore, *NCAM1* annotation (rs2303377) was reported to have an association [Genotype TT] with increased response to duloxetine in people with Major Depressive Disorder [compared to genotypes CC + CT]. The PGx impact of *NCAM1* may be a potential prediction for future works on an inner network among *DRD2*, *NCAM1*, *LINGO1*, *DRD3*, *COMT*, and *MAPT* [see Figure 9].

### 3.5. Expression Analysis

**Figure 10:**
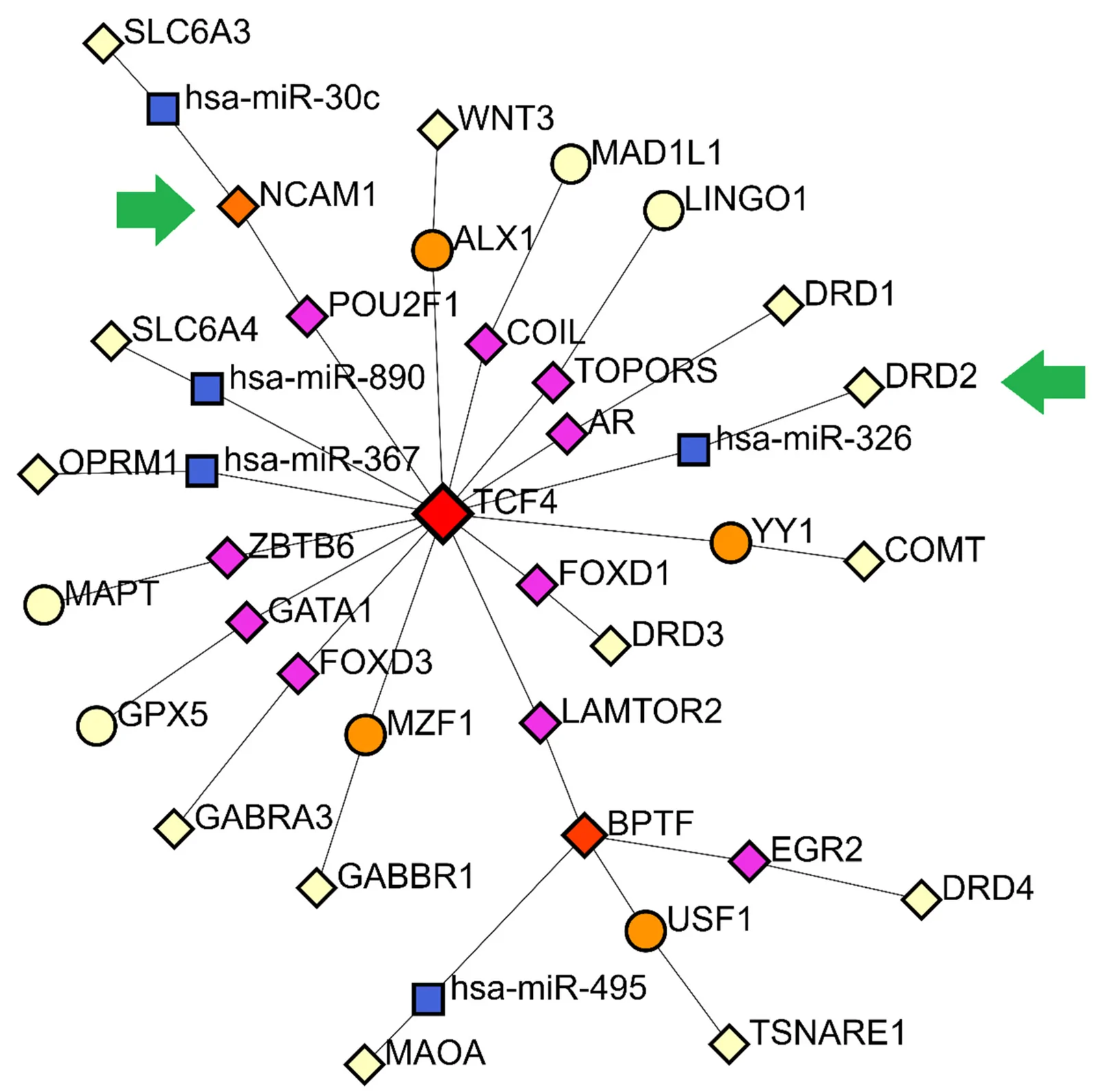
Fruchterman–Rheingold model of FGL members set based on TF-miRNA coregulatory interactions through NetworkAnalyst tool (ver. 3). The green arrows highlight focused predictions in the previous step regarding DRD2 and NCAM1 interactions.

TF-miRNA coregulatory interactions by NetworkAnalyst (R-based online tool) [73] were performed for more investigations on more complicated interactions among the FGL members in regulatory levels governed by miRNAs and transcription factors (TFs). In a Fruchterman–Rheingold model, a regulatory network with TCF4 as a key hub gene was found. The predictions of the current analysis confirmed the prior findings in STRING-MODEL and indicated that DRD2 can be linked to TCF4 by hsa-miR-326, while NCAM1 can be linked to TCF4 by POU2F1 (Figure 10). Thus, in this specific case, a miRNA and a TF could successfully fill the gaps of functional predictions and strengthen the finding with the GWAS fine-mapping tool.

## 4. Discussion

The present study integrates large-scale GWAS fine-mapping with systems biology to find more details about the molecular architecture underlying PTSD. Rather than identifying a single dominant locus or pathway, the findings reinforce PTSD as a polygenic, network-driven condition characterized by convergent disruption across dopaminergic signaling, synaptic plasticity, neurodevelopmental regulation, and neuroimmune interaction. This system-level architecture is consistent with clinical observations of marked heterogeneity in PTSD symptom expression, comorbidity, and treatment response.

Fine-mapping of genome-wide significant variants predicted multiple loci distributed across the genome, with no single region accounting for the observed risk signal. Instead, clustering of variants within genes such as *FOXP2*, *TCF4*, *NCAM1*, *LINGO1*, *MAPT*, and dopaminergic pathway genes, underscores the importance of transcriptional control, neuronal connectivity, and synaptic modulation in PTSD vulnerability. The prominence of *FOXP2*, traditionally associated with cortico-striatal circuit development, synaptic plasticity, and adaptive learning, suggests that susceptibility to trauma-related psychopathology may be shaped in part by pre-existing differences in neural network organization. Similarly, *TCF4*, identified here as a central regulatory hub in transcription factor–miRNA network analyses, is known to influence neuronal differentiation, excitability, and large-scale network coordination, positioning it as a plausible integrator of stress, reward, and cognitive control circuitry. Genes such as *NCAM1*, involved in synaptic adhesion and plasticity, and *LINGO1*, associated with axonal growth inhibition and myelination, further implicate distributed connectivity and plasticity mechanisms, rather than isolated dysfunction within canonical fear circuits alone.

A central observation of this work is that GWAS-derived candidate genes, when examined in isolation, formed a sparse and weakly connected protein–protein interaction network. However, the integration of established RDS-related genes resulted in a highly interconnected functional network. This output does not imply causality but introduces mechanistic plausibility that dopaminergic homeostasis genes may act as stabilizing nodes that bridge otherwise-fragmented genetic risk signals. The emergence of a coherent network incorporating *DRD1–4*, *COMT*, *MAOA*, *SLC6A3*, *SLC6A4*, *OPRM1*, and GABAergic receptors, measured by the GARS test, strengthened the hypothesis that reward circuitry dysfunction represents a common biological denominator linking PTSD with its frequent comorbidities, including substance use disorders, affective dysregulation, and impaired stress-coping.

Regulatory network analyses further strengthened this interpretation by predicting *TCF4* as a key transcriptional hub, with miRNA- and transcription factor-mediated connections linking *DRD2* and *NCAM1*. These predictions offer a plausible regulatory mechanism through which genetic risk variants may influence dopaminergic tone, synaptic connectivity, and stress responsivity. Importantly, this regulatory convergence aligns with emerging models that conceptualize PTSD as a disorder of maladaptive salience attribution and network instability, rather than a condition confined to fear circuitry alone.

From a translational perspective, pharmacogenomic annotation of the Final Gene List revealed that all canonical RDS-related genes were indicated to have at least one pharmacogenomic association, whereas only a minority of non-RDS GWAS-derived genes showed similar annotations. While exploratory, this asymmetry suggests that dopaminergic and reward-related genes may be disproportionately actionable in stratified treatment frameworks, even when they are not the most statistically significant loci in association analyses. This observation does not diminish the relevance of non-dopaminergic pathways, but highlights the importance of integrating functional and regulatory relevance alongside statistical association when prioritizing therapeutic targets [74].

### 4.1. Limitations

Several methodological considerations warrant acknowledgment. The fine-mapping strategy relied on GWAS summary statistics and reference-panel-based linkage disequilibrium estimates rather than individual-level genotype data, limiting the ability to perform conditional analyses or definitively assign causality to specific variants. Approximate Bayesian fine-mapping and posterior inclusion probabilities were therefore interpreted as probabilistic indicators rather than definitive causal assignments, particularly under the assumption of a single causal variant per locus. In addition, the GWAS Catalog aggregates studies with heterogeneous PTSD phenotypes, trauma exposures, diagnostic instruments, and comorbidity profiles, which may obscure subtype-specific genetic architecture. Differences in ancestry composition across contributing studies, coupled with reliance on reference LD panels, may further influence locus resolution and generalizability, which, in turn, reflect the reduced specificity, and can be solved by selecting the same GWAS datasets from a specific ancestry in the future works. Finally, the incorporation of RDS-related genes into network analyses was hypothesis-driven and exploratory, intended to test whether dopamine homeostasis genes plausibly bridge sparse GWAS-derived interaction networks rather than assert causal primacy [75]. It should be noted that the manuscript included references to GARS and KB220 in a manner that may be interpreted as endorsement, despite declared intellectual property interests; thus, data-driven findings should be considered separated from proprietary or hypothesis-driven frameworks.

### 4.2. Future Studies

Taken together, these findings support a reconceptualization of PTSD as a disorder of distributed neurobiological network instability, with dopaminergic and reward-related pathways functioning as integrative nodes linking genetic risk, stress exposure, neuroimmune modulation, and clinical expression. Future studies should prioritize replication in harmonized cohorts with refined phenotyping, including symptom clusters, trauma type, and longitudinal outcomes [76]. Integration of fine-mapped loci with brain-specific expression quantitative trait loci and epigenomic datasets may further elucidate regulatory mechanisms linking genetic variation to functional brain changes. Experimental interrogation of key regulatory hubs—particularly those involving TCF4, NCAM1, and dopaminergic pathway genes—may help define actionable targets. Ultimately, combining genetic risk profiles (e.g., GARS) with neuroimaging, neuroimmune markers, and pharmacogenomic annotation may advance precision-oriented stratification strategies for trauma-exposed populations while avoiding reductionist interpretations of PTSD as a single-pathway disorder [77].

Within this framework, the present findings provide a biological context for understanding why PTSD manifests with such marked heterogeneity and why uniform treatment approaches often yield variable results. By situating genetic risk [78] within interacting functional and regulatory systems relevant to stress, reward, and adaptation, this work helps frame PTSD as a neurophysiological condition shaped by network-level vulnerability rather than a single-pathway disorder, thereby setting the stage for the concluding synthesis [79].

### 4.3. Policy Statement

Post-Traumatic Stress Disorder (PTSD) represents a substantial and persistent public health burden, particularly among military, first-responder, and trauma-exposed populations. Despite extensive investigation, prevailing prevention and treatment paradigms remain largely reactive, symptom-focused, and insufficiently aligned with underlying neurobiological heterogeneity. The findings presented in this work support a policy evolution toward precision neurobiological management, capable of informing risk stratification, prevention, and treatment for PTSD.

Convergent evidence from large-scale genomics, neuroimaging, neuroimmunology, and systems biology indicates that PTSD is not a unitary disorder but a spectrum of stress-related neurobiological phenotypes. Dysregulation of dopaminergic reward circuitry (e.g., Reward Deficiency Syndrome), stress-responsive neuroendocrine systems, immune and inflammatory signaling, and epigenetic modulation appear to interact dynamically, particularly in individuals with inherited vulnerability who are exposed to repeated or severe trauma. Continued reliance on broadly suppressive pharmacologic strategies—especially those that further impair dopaminergic tone—may inadequately address, or potentially exacerbate, long-term dysfunction in biologically vulnerable subgroups.

From a policy standpoint, these findings support several actionable priorities:

1. Early Risk Identification and Stratification

Trauma-exposed individuals in high-risk occupational settings may benefit from ethically governed, voluntary approaches to early neurobiological risk assessments, integrating genetic, neuroendocrine, and immune markers. Such strategies are intended to support prevention and targeted intervention rather than stigmatization.

2. Precision-Informed Preventive Strategies

Interventions aimed at restoring neurobiological homeostasis—particularly within reward and neuroimmune systems—merit systematic evaluation as complements to established psychological and behavioral therapies. Deployment during defined windows of vulnerability (e.g., post-deployment or post-acute trauma exposure) may reduce progression to chronic illness.

3. Integration of Objective Neurobiological Endpoints

Future clinical trials and care models should incorporate objective neurobiological endpoints, including neuroimaging connectivity measures, immune markers, and pharmacogenomic profiles, alongside symptom-based outcomes to better characterize treatment response heterogeneity.

4. Avoidance of One-Size-Fits-All Pharmacotherapy

Policy guidance should discourage the indiscriminate long-term use of agents that suppress adaptive stress and reward circuitry without addressing underlying neurobiological deficits. Personalized approaches aligned with individual neurogenetic and neuroimmune profiles may improve both efficacy and safety.

5. Translational Pilots and Cross-Agency Collaboration

Coordinated efforts among research institutions, public health agencies, and defense and veterans’ health systems are warranted to support translational pilot programs evaluating precision-based PTSD prevention and treatment models.

Within this umbrella of precision neurobiological management, genetic risk stratification tools (e.g., the Genetic Addiction Risk Score or GARS) have accumulated sufficient convergent evidence to support their use in research and prevention-oriented stratification frameworks. Pro-dopaminergic regulatory compounds (e.g., KB220), which have been explored as adjuncts to established care pathways, warrant further independent clinical validation.

In sum, PTSD policy must evolve from late-stage symptom management toward early, biology-informed prevention and intervention. Advances in genomics, neuroimaging, and biology systems now make such an approach scientifically plausible. Responsible integration of these tools has the potential to reduce long-term disability, improve functional recovery, and align PTSD care with the principles of modern precision medicine. This aim, in part, may be accomplished by early genetic testing (e.g., GARS and addition of other candidate genes discovered herein).

## 5. Conclusions

PTSD can be understood as a condition involving multiple neurobiological systems. This may be embodied as the dysregulation of neural, immune, endocrine, genetic, and metabolic pathways. Findings from preclinical models and human studies consistently implicate maladaptive stress responses, characterized by alterations in prefrontal regulation circuitry, altered HPA axis feedback, immune and inflammatory responses, and dopaminergic abnormalities related to salience and reward. Large neuroimaging, genomic, and epigenetic studies reveal significant heterogeneity. Distinct neurobiological profiles may predict variability in symptomology, treatment response, and comorbidity. These findings may support a shift toward the incorporation of a neurophysiological conceptualization of PTSD risk profiles. Such approaches hold promise for improving early risk identification, guiding personalized interventions, and accelerating the development of targeted preventive and therapeutic strategies for trauma-exposed populations.

As an original article, this presentation of research may be limited by potential selection bias and does not provide quantitative effect size estimates. Additionally, our episodic analyses on GWAS dataset of PTSD indicated that GARS genes can potentially describe the gaps between the GWAS fine-mapping results and systematic interactions of functional and regulatory players in the cellular and molecular levels of PTSD, which may lead to uncovering more about the mechanism of action and precision treatment of this manifestation.

## 6. Patents

Dr. Blum owns all patents domestic and foreign-issued or pending patents related to both GARS and KB220.

## Data Availability

Any further data including Python codes will be available on a reasonable request from the corresponding author via email.

## Abbreviations

The following abbreviations are used in this manuscript:

PTSD	Post-Traumatic Stress Disorder
GWAS	Genome-Wide Association Studies
LD	Linkage Disequilibrium
PGx	Pharmacogenomics
RDS	Reward Deficiency Syndrome
PBM	Precision Behavioral Management
SUD	Substance Use Disorder
NIDA	National Institute on Drug Abuse
GABA	Gamma-aminobutyric acid
CUD	Cocaine Use Disorder
AUD	Alcohol Use Disorder
ADHD	Attention-Deficit/Hyperactivity Disorder
DMN	Default Mode Network
GARS	Genetic Addiction Risk Score
CNS	Central Nervous System
SCID	Severe Combined Immunodeficiency
CRF	Corticotropin-releasing factor
POMC	Proopiomelanocortin
EOP	Endogenous Opiate Peptides
ACTH	Adrenocorticotropic Hormone
ConA	Concanavalin A
IBD	Irritable Bowel Disorder
rsfMRI	resting-state Functional Magnetic Resonance Imagining
LGMM	Latent Growth Mixture Modeling
NE	Norepinephrine
DOD	Department of Defense
CISM	Critical Incident Stress Management
WHO	World Health Organization
ORs	Odds Ratios
CIs	Confidence Intervals
SE	Standard Error
QQ	Quantile–Quantile
PIP	Posterior Inclusion Probability
